# The Impact of Sports Injuries on the Academic Performance and Mental Health of High School Students in Jeddah

**DOI:** 10.7759/cureus.73912

**Published:** 2024-11-18

**Authors:** Alwaleed R Shata, Aya R Shata, Dania F Bogari, Turki Y Alhazzazi

**Affiliations:** 1 Medicine, AS Sports Medicine Club, American International School of Jeddah, Jeddah, SAU; 2 Endodontics, Faculty of Dentistry, King Abdulaziz University, Jeddah, SAU; 3 Oral Biology, Faculty of Dentistry, King Abdulaziz University, Jeddah, SAU

**Keywords:** academic performance, high school, mental health, sports injuries, sports medicine

## Abstract

Objectives

This study aimed to investigate the effects of sports injuries on the academic performance and mental health of high school students in Jeddah.

Materials and methods

This cross-sectional study utilized a closed-ended electronic questionnaire (e-questionnaire) sent to students from four international high schools in Jeddah. The e-questionnaire included questions assessing the level of academic courses, the frequency and types of sports injuries encountered, and the impact of these injuries on both academic performance and mental health. The chi-square test was used to establish relationships between categorical variables, with a p-value threshold of <0.05 determining statistical significance.

Results

Of the 117 high school students who participated in the study, 56.4% (n=66) were male and 43.6% (n=51) were female. Approximately 83.8% (n=98) of students engaged in sports. Interestingly, 65.0% (n=76) reported experiencing a sports injury during their high school years, with 73.7% (n=56) of these injuries occurring during school-related activities. These sports injuries significantly affected students' academic performance and mental health, impacting 65.8% (n=50) and 76.1% (n=51) of respondents, respectively. Major consequences included delayed assignments (96%, n=48 ), missed exams (88%, n=44), suicidal thoughts (56.9%, n=29), and self-harm thoughts (23.5%, n=12). Surprisingly, only 25.4% (n=17) of students received support from their schools. Furthermore, when support was provided, it was primarily in the form of medical assistance, with a notable lack of mental health support.

Conclusion

Our study revealed a significant gap in the school support system for students, which requires urgent attention. High school students are highly active and often experience sports-related injuries, particularly during school activities. These injuries can profoundly affect students' academic performance and mental health. Therefore, the findings of our study are essential for raising awareness among students, parents, and teachers about sports injury management. Additionally, although our study focused solely on private international schools, it is crucial for stakeholders in both government and private education sectors to address this issue at a broader level, ensuring adequate support for students facing such injuries. This will help protect the well-being and safety of future students.

## Introduction

Playing sports regularly is vital for maintaining both physical and mental health, especially during adolescence [1,2]. However, this participation comes with costs and consequences when unfortunate injuries occur. While several studies have shown that sports injuries can negatively impact both the physical and mental health of athletes, potentially leading to catastrophic outcomes if not managed appropriately, limited research has focused on the effects of such injuries during adolescence [3-5]. Therefore, this study aimed to investigate the effects of sports injuries on the academic performance and mental health of high school students in Jeddah.

## Materials and methods

This cross-sectional study utilized an English-language closed-ended electronic questionnaire (e-questionnaire) distributed to students from four international high schools in Jeddah, Saudi Arabia. The e-questionnaire included items assessing academic course levels, the frequency and types of sports injuries encountered, and the impact of these injuries on both academic performance and mental health. The questionnaire was developed and reviewed by three experts in sports injury, mental health, and survey design to ensure clarity and validity. We piloted the initial version with 25 participants to evaluate the clarity of the questions. Based on their feedback, the questionnaire was revised, and the final version was sent to students from the four international high schools in Jeddah.

The data for this study were analyzed using IBM SPSS version 27 (Armonk, NY: IBM Corp.). Descriptive statistics were employed to summarize the data as follows: frequencies and percentages were used for categorical variables, while means and standard deviations were calculated for continuous variables. The relationships between categorical variables were assessed using the chi-square test, which helped determine if there were statistically significant associations between variables such as gender, academic course enrollment, sports participation, and injury outcomes. A p-value threshold of <0.05 was used to determine statistical significance. Results with p-values below this threshold led to the rejection of the null hypothesis, indicating that the observed associations were unlikely to have occurred by chance. This approach ensured that the analysis effectively captured the patterns and relationships between sports participation, injuries, academic performance, and mental health impacts among the high school students in the study. The e-questionnaire also included a section for written informed consent, which was obtained from all participants and adhered fully to the World Medical Association Declaration of Helsinki.

## Results

Demographics of the study sample

This questionnaire was distributed to students from four different international schools in the city of Jeddah, Saudi Arabia. Of the 117 high school students who participated in the study, 56.4% (n=66) were male and 43.6% (n=51) were female (Table 1). Most participants were in 12th grade (44.4%, n=52), followed by 11th grade (26.5%, n=31). A smaller portion was in the ninth and 10th grades, 15.4% (n=81) and 13.7% (n=16), respectively (Table 1). In terms of course enrollment, 57.3% (n=67) of students were enrolled in Advanced Placement (AP) courses, 17.9% (n=21) in International Baccalaureate (IB) courses, and 41.9% (n=49) in regular courses (Table 1).

**Table 1:** Demographics of the study participants. ^a^Multiple answer questions, please don’t add counts and percentages. AP: Advanced Placement; IB: International Baccalaureate

Demographics		Count	%
Total		117	100.0
Gender	Male	66	56.4
	Female	51	43.6
Grade level	9th grade	18	15.4
	10th grade	16	13.7
	11th grade	31	26.5
	12th grade	52	44.4
Are you enrolled in regular academic courses or advanced courses?^a^	AP	67	57.3
	IB	21	17.9
	Regular	49	41.9

Sports participation and injury-related experience

**Figure 1:**
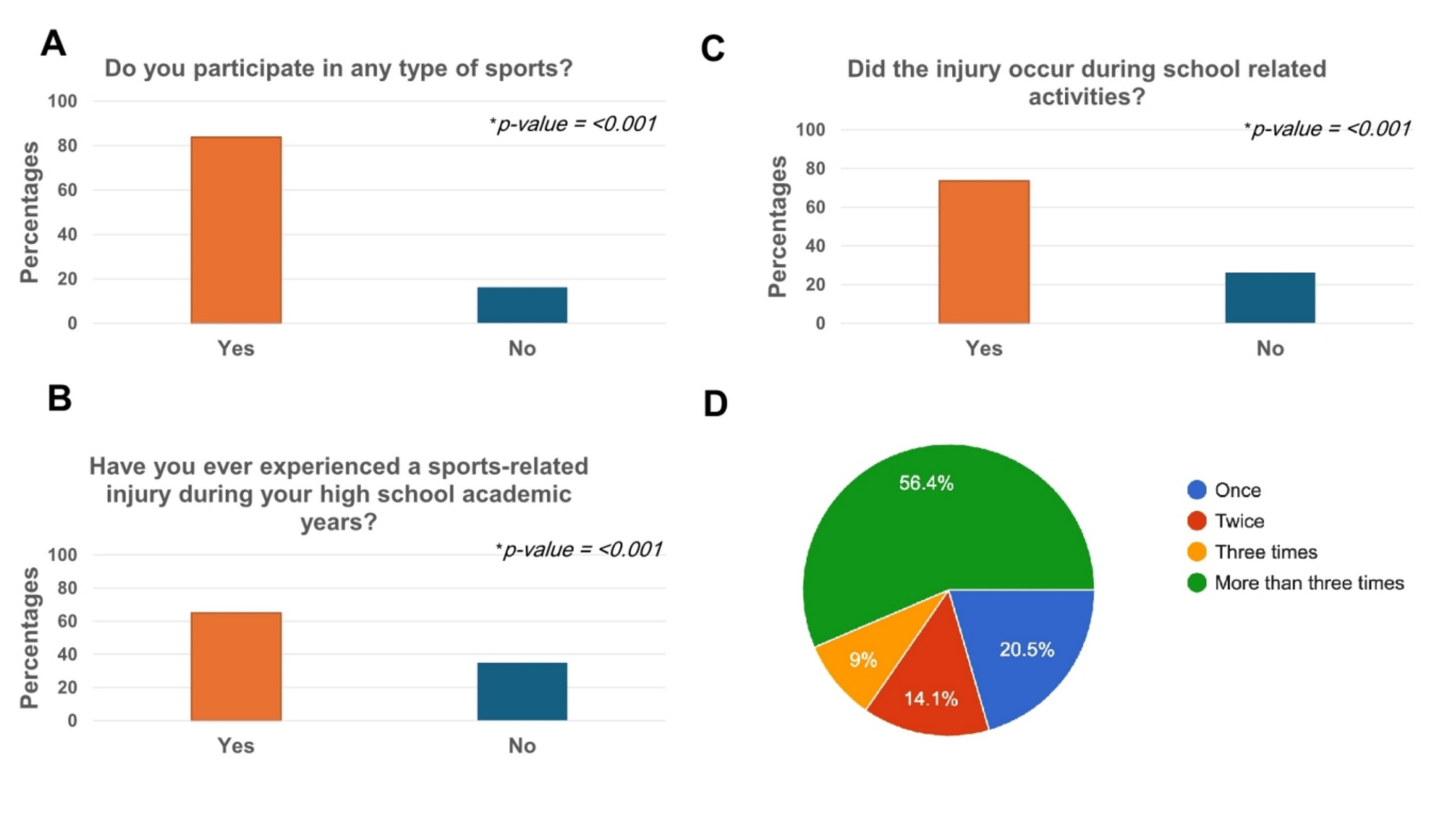
Sports participation and injury-related experiences. (A) Percentage of participants engaged in various types of sports. (B) Percentage of sports injuries among participants during high school academic years. (C) Injuries sustained during sports-related activities. (D) Frequency of injuries.

About 83.8% (n=98) of students participate in sports (Figure 1, panel A). A significant relationship exists between gender and participation in sports (p<0.001), with males (64.3%, n=63) significantly more likely to participate than females (35.7%, n=35), indicating a gender disparity in sports involvement (Table 2). Additionally, there is a significant association between gender and sports injuries (p<0.001), with males (69.7%, n=53) experiencing more injuries than females (30.3%, n=23) (Table 2). Furthermore, the number of injuries increased with grade level, with 12th-grade students showing the highest rates of injury (55.3%, n=42) (p<0.001) (data not shown).

**Table 2:** Gender correlations in regards to e-questionnaire responses. ^a^The chi-square test level <0.05 was considered significant.

Variables		Total	Gender		p-Value
			Male	Female	
Do you participate in any type of sports?	Yes	98	63 (64.3%)	35 (35.7%)	<0.001^a^
	No	19	3 (15.8%)	16 (84.2%)	
Have you ever experienced a sports-related injury during your high school academic years?	Yes	76	53 (69.7%)	23 (30.3%)	<0.001^a^
	No	41	13 (31.7%)	28 (68.3%)	
If yes, how many times have you experienced a sports injury?	Once	14	6 (42.9%)	8 (57.1%)	<0.001^a^
	Twice	11	4 (36.4%)	7 (63.6%)	
	Three times	7	5 (71.4%)	2 (28.6%)	
	More than three times	44	38 (86.4%)	6 (13.6%)	
Did the injury occur during school-related activities?	Yes	56	45 (80.4%)	11 (19.6%)	<0.001^a^
	No	20	8 (40.0%)	12 (60.0%)	
Did the injury affect your academic performance at school?	Yes	50	40 (80.0%)	10 (20.0%)	0.007^a^
	No	26	13 (50.0%)	13 (50.0%)	
Did the injury affect your mental health?	Yes	51	40 (78.4%)	11 (21.6%)	0.201
	No	16	10 (62.5%)	6 (37.5%)	
Did you receive any support from your school following the injury?	Yes	17	8 (47.1%)	9 (52.9%)	0.002^a^
	No	50	42 (84.0%)	8 (16.0%)	

**Figure 2:**
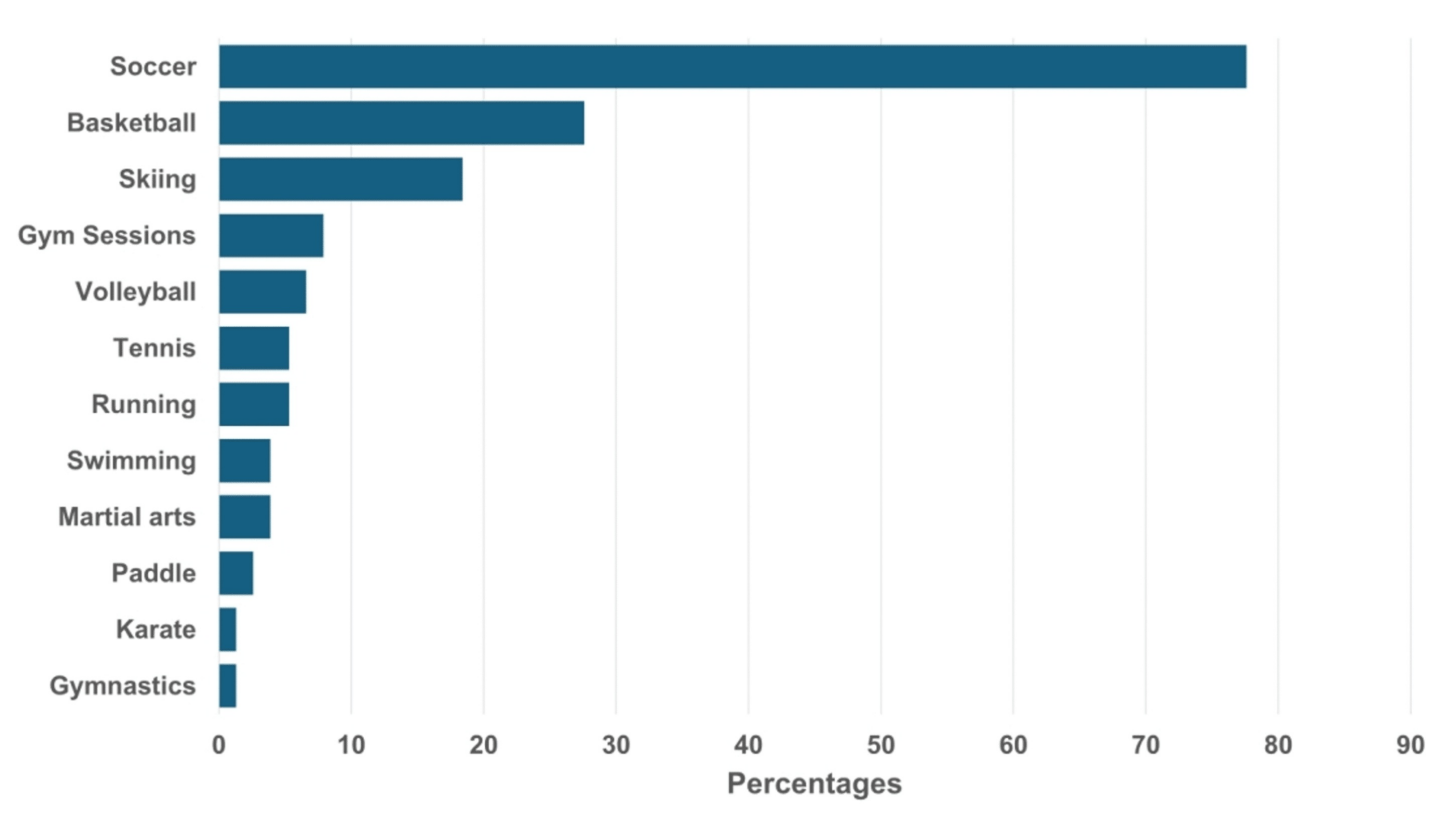
Types of sports-related injuries during high school academic years.

Approximately 65.0% (n=76) of the students reported experiencing a sports injury during their high school academic years (Figure 1, panel B). Notably, 73.7% (n=56) of these injuries occurred during school-related activities (Figure 1, panel C). Additionally, 56.4% (n=44) reported sustaining more than three injuries during their high school years (Figure 1, panel D). Soccer was associated with the highest percentage of injuries (77.6%, n=59), followed by basketball (27.6%, n=21) and skiing (18.4%, n=14) (Figure 2).

The impact of sports injury on students' academic performances

**Figure 3:**
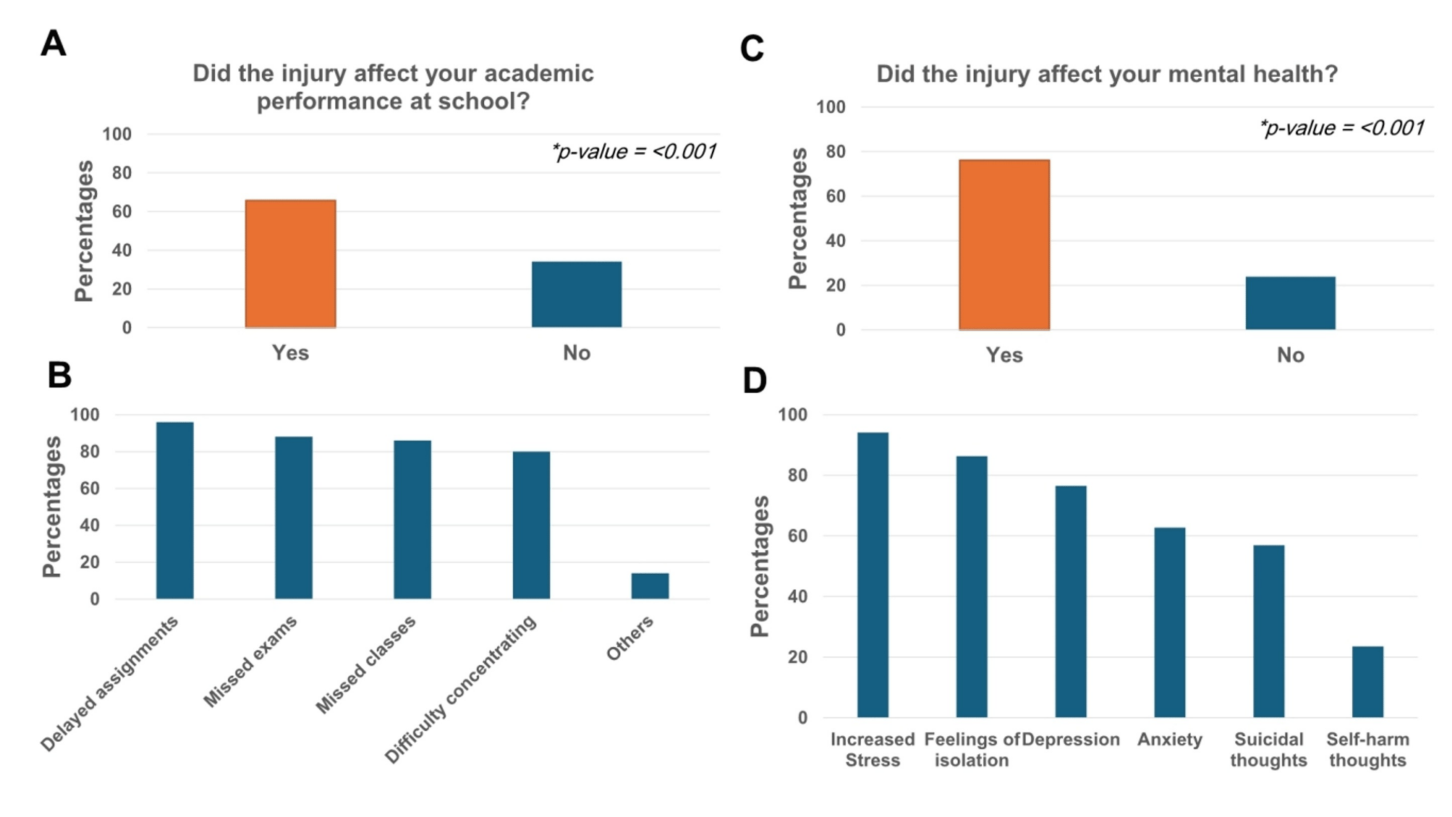
The impact of sports injuries on academic performance and mental health. (A) Academic performance. (B) The extent of injuries' effects on academic performance. (C) Mental health. (D) The extent of injuries' effects on mental health.

Of the participants, 65.8% (n=50) reported that sports injuries negatively affected their academic performance (p<0.001) (Figure 3, panel A). This difference was also statistically significant between genders, with males tending to be affected more than females (Table 2). Among those impacted, the most common effects were delayed assignments (96%, n=48), followed by missed exams (88, n=44%), missed classes (86, n=43%), and difficulty concentrating (80%, n=40) (Figure 3, panel B).

The impact of sports injury on students' mental health

Of the participants, 76.1% (n=51) of students with injuries reported that the injury affected their mental health (p=0.001) (Figure 3, panel C). However, unlike academic performance, sports injuries affected both genders equally, with no statistically significant difference (Table 2). Mental health issues included increased stress (94.1%, n=48), feelings of isolation (86.3%, n=44), depression (76.5%, n=39), anxiety (62.7%, n=32), suicidal thoughts (56.9%, n=29), and thoughts of self-harm (23.5%, n=12) (Figure 3, panel D).

School involvement in help and support of students encountering sports injury

**Figure 4:**
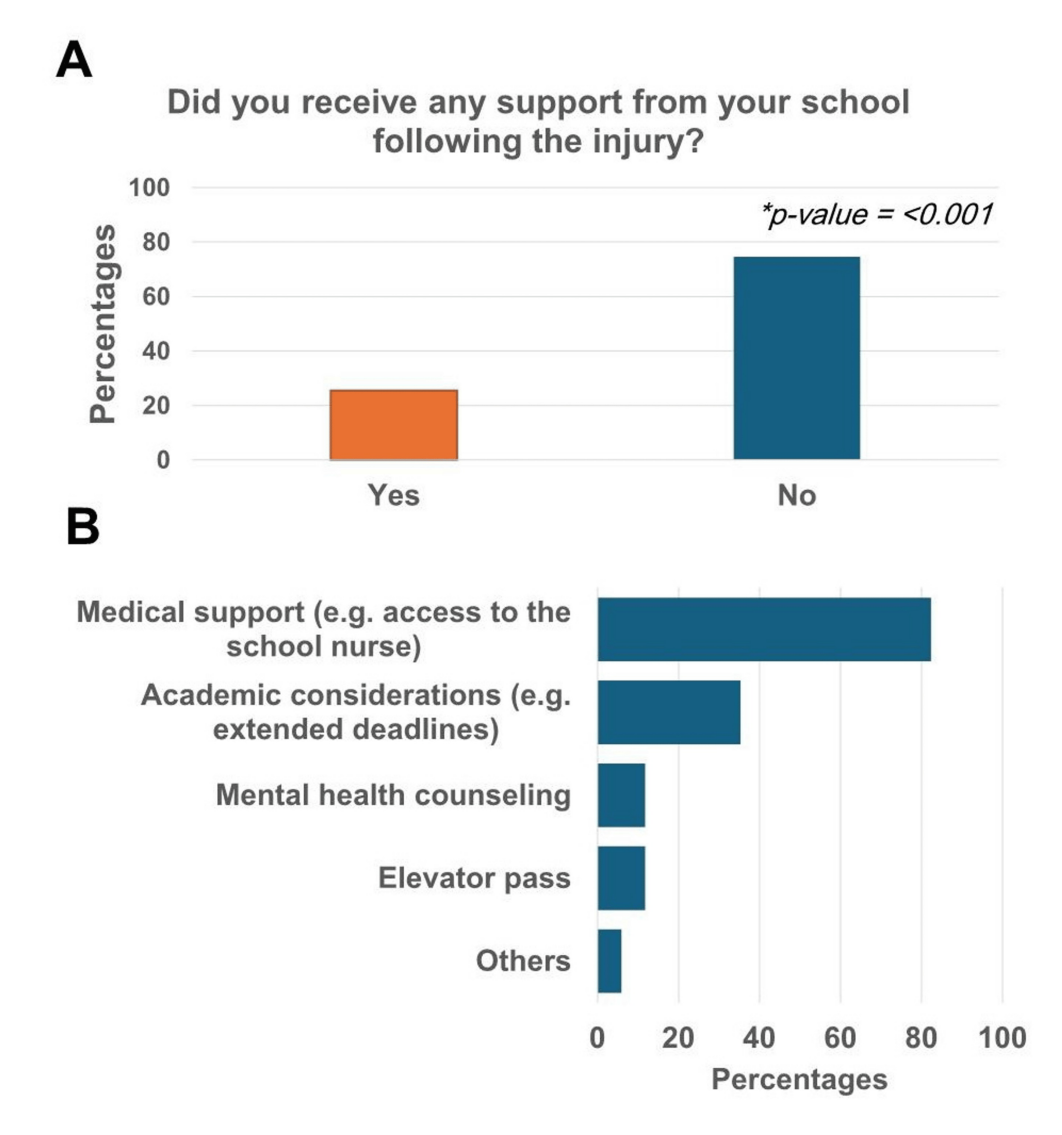
School support for injured students. (A) Percentage of students receiving support from their schools. (B) Types of support received.

Only 25.4% (n=17) of students received post-injury support from their schools (p<0.001) (Figure 4, panel A). The most common type of support was medical assistance (82.4%, n=14), while some students received academic consideration (35.3%, n=6), and a few received mental health counseling (11.8%, n=2) (Figure 4, panel B).

## Discussion

The extensive impact of sports injuries on adolescent students has been inadequately studied [3,5-7]. Therefore, this study aimed to investigate the effects of sports injuries on the academic performance and mental health of high school students in Jeddah.

Our results revealed that a significant number of students are engaged in sports (83.8%). However, a significant percentage of these students (65%) experienced a sports injury during their high school years, with a substantial number reporting more than three such injuries (56%). Notably, 73.3% of these injuries occurred during school-related activities (Figure 1, panels A-D). Soccer appears to be the most common cause of school sports injuries, followed by basketball (Figure 2). Our findings are consistent with a study conducted among professional sports clubs in Riyadh, which also identified soccer as the sport associated with the highest incidence of major injuries [8].

This underscores the important role schools play in preventing and supporting students who encounter such injuries. However, our results highlighted a significant gap in the school system in Jeddah regarding the proper management of students with sports-related injuries. It is worth noting that the students who participated in this questionnaire were from international schools, which are considered to serve high socioeconomic status students who should receive the highest levels of educational support, as well as both physical and mental health support. The low percentage of school support for students with sports injuries (25%) is alarming, especially given that these injuries occurred during school-related activities. Additionally, when support was provided, it was mostly in the form of medical assistance, with a notable lack of mental health support, indicating a significant gap in post-injury care within the school-based support system (Figures 4A, 4B). Studies have shown that adolescents often experience a wide range of psychological issues following a sports-related injury, such as feelings of shame and harmful behaviors [3]. Similarly, our results indicate that the majority of students suffered from increased stress (94.1%), feelings of isolation (86.3%), and depression (76.5%). Shockingly, a high percentage of students reported suicidal thoughts (56.9%) and self-harm thoughts (23.5%) (Figure 3, panel D). This highlights the importance of providing mental health support on par with physical health support following sports-related injuries, starting in the school setting.

In contrast to our findings, some studies suggest that gender can be a risk factor for mental health issues after a sports injury, with females tending to suffer more than males [3,9]. However, in our study, gender did not have a significant effect on mental health following injury, which may be attributed to the small sample size. This point may need further investigation within our community.

On the other hand, academic performance appears to be significantly affected after sports injuries, though this aspect has been poorly studied in the literature [6]. Our results indicate that students’ academic performance is negatively impacted after sports injuries (65.8%). This impact includes delayed assignments, missed classes, and exams. These findings align with other studies that reported poor grades and absenteeism as major concerns following sports injuries [6,7]. Interestingly, the majority of students (99.1%) agree that sports improve academic performance and mental health (data not shown). This is an encouraging percentage, despite the several injuries they encountered, as mentioned previously (Figure 1, panel D).

Finally, our study has some limitations. One notable limitation is the small number of responses, which may be attributed to a loss of interest among students in participating in scientific questionnaires. Additionally, we intended to include more questions to cover other relevant aspects. However, considering the students' age, we believed that longer questionnaires might result in a lower response rate compared to shorter ones. Moreover, we should also appreciate the potential students' self-reporting bias.

## Conclusions

Our study revealed a significant gap in the school support system for students, which requires urgent attention. High school students are highly active and often experience sports-related injuries, particularly during school activities. These injuries can profoundly affect students' academic performance and mental health. Therefore, the findings of our study are essential for raising awareness among students, parents, and teachers about sports injury management. Additionally, although our study focused solely on private international schools, it is crucial for stakeholders in both government and private education sectors to address this issue at a broader level, ensuring adequate support for students facing such injuries. This will help protect the well-being and safety of future students.
